# One size does not fit all: adapt and localise for effective, proportionate and equitable responses to COVID-19 in Africa

**DOI:** 10.1136/fmch-2020-000709

**Published:** 2021-04-02

**Authors:** Hayley MacGregor, Melissa Leach, Akhona Tshangela, Tabitha A Hrynick, Shelley Lees, Eva Niederberger, Melissa Parker, Santiago Ripoll Lorenzo, Hana Rohan, Megan Schmidt-Sane, Olivia Tulloch, Annie Wilkinson

**Affiliations:** 1 Institute of Development Studies, Brighton, UK; 2 Africa Centres for Disease Control and Prevention, African Union, Addis Ababa, Ethiopia; 3 Department of Global Health and Development, London School of Hygiene & Tropical Medicine, London, UK; 4 Anthrologica, Oxford, UK

**Keywords:** health policy, public health, social determinants of health, global health

## Introduction

The heterogeneous epidemiological picture for COVID-19 in Africa continues to generate debate. Modelling projections raise speculation about the phases and trends of SARS-CoV-2 outbreaks across the continent and how these differ from outbreaks elsewhere.^1–4^ Continental efforts drew praise in the first wave of COVID-19, and success has been linked to African experience of epidemics and to decisive leadership.^4^ Yet the tendency at the outset of the pandemic with initial responses to the threat of COVID-19 was for African governments to look to standard models emphasising central control, following the WHO and partly mirroring the stringent restrictions as already deployed in Asia and Europe. The negative effects of ‘lockdowns’ and a vertical, disease-specific ‘health security’ response on livelihoods, food security and healthcare for other conditions have threatened to overshadow the direct suffering and even mortality engendered by COVID-19 alone.^5^ These effects have been evident also in high-income countries but manifested rapidly and intensely in Africa. This scenario highlights the problems of generic approaches to response given very different settings in which a pandemic unfolds, suggesting that further attention should have been paid by decision makers to significant adaptation to African realities from the start. The continent is also hugely diverse in health system and social protection capacities, demographics and population density, underscoring an argument for adaptation and contextualised responses across African settings. Moreover, since the initial imposition of extensive curtailment, political and economic pressures necessitated an easing of ‘lockdowns’ as 2020 progressed. More limited measures are evident in situations where cases of community transmission of SARS-CoV-2 and deaths have risen as some countries face a second wave.^6^ All of these factors, alongside the paucity of resources, lack of definitive pharmacological treatments and likely delays in deployment of vaccines on the continent, have further intensified the need for context-appropriate public health and social measures, tailored to ensure proportionality of responses as differing scenarios unfold.

We contend that a critical part of adaptation and proportionality is a localisation of response that builds on people’s own inventiveness and the knowledge and experience of local organisations. Drawing on knowledge exchange webinars run collaboratively between the Africa Centres for Disease Control and the Social Science in Humanitarian Action Platform in 2020, we consider positive examples of adaption that have emerged. We present a preliminary typology of important domains for localisation of public health and social measures, with a focus on interventions to protect people who are vulnerable—socially, economically, politically and also biologically. We call for acknowledgement of the importance of local-level responses and the need to support these and for the establishment of fora to share learning about adaptation and effective models. Streamlined funding mechanisms that allow for rapid flow of resources to support initiatives ‘from below’^7^ also need to be strengthened.

### The politics of easing restrictions: balancing public health action against the impacts of measures

The current dilemmas faced by African governments—as elsewhere—bring to the fore the reality that epidemic preparedness and response are not neutral, scientific processes but deeply political and social as well. Decision makers are inevitably influenced by politics and economics, in addition to scientific data. Indeed, amidst the great scientific and societal uncertainty that an emergent pathogen generates, it may be politically easier to pursue standardised routes of preparedness and response, which prioritise containment through top-down, disease-specific, state-led interventions.^8^ The political responses across African countries have been complex and diverse and reflect national and geopolitical tensions, as elsewhere. Definitive ‘lockdowns’ can have political meaning and even gain for governments that wish to be seen to act swiftly and take strong control in a crisis. In the extreme, some have been accused of using the pandemic to legitimate excessively authoritarian responses.^9^ Stringent measures might have delayed outbreaks initially or even contained them in some contexts, but the secondary impacts on economies, livelihoods and health have necessitated a weighing up of the relative effects of an outbreak surge against those of the restrictions. These decisions have become even more difficult as several African countries have experienced a second wave of COVID-19 that has proven to be more deadly than the first and as mutations have increased transmissibility of the virus.

In May, Africa CDC issued guidance on the easing of restrictions while warning also that it was likely Africa would yet become a frontier of the pandemic^10^ with community transmission increasing in some countries. These apparently paradoxical messages highlighted that such easing, too, involved consideration of political and economic factors as well as epidemiological ones—the lifting of lockdowns in Africa became necessary to save lives, economies and livelihoods, as was made explicit in the launch of an African Union campaign ‘Africa against COVID-19’ in mid-August 2020. Social and economic vulnerability on the continent is exacerbated by high reliance on informal economic activity and on local markets for food, a lack of social protection safety nets^11^ and living conditions in densely populated and poorly serviced urban settlements where physical distancing, isolation and hygiene measures are difficult to achieve.^12^ Sustaining livelihoods is ultimately linked to saving lives but easing restrictions also saves lives more directly. In addition to food insecurity causing malnutrition, the prioritisation of responses to COVID-19 has disrupted essential health services, such as for immunisation and care of other prevalent diseases. Contributing factors include issues of supply (disruption of flows of drugs and equipment, illness or redeployment of staff) and access (lack of transport to reach services and fear of breaching restrictions).^13^

### One size does not fit all: adaptation and localisation of responses is key

These realities point to a strong argument for responses that are adapted and proportionate to local contexts and epidemiology. With the easing of more extreme containment measures, national-level interventions for reducing community transmission that minimise impact on broader aspects of socioeconomic well-being have become more important. At local levels, it has become necessary to adapt physical distancing, hand washing, isolation and community-based care to prevalent realities such as multi-occupancy in crowded dwellings or the absence of running water.

As restrictions ease for general publics, a further key question is how to protect those particularly vulnerable to COVID-19. Strategies may include developing practicable means for ‘shielding’ the clinically vulnerable^14^ in multigenerational households, medication delivery for the chronically ill to reduce exposure or food and cash relief for those who are suffering economically.

In this respect, initiatives developed at community level, led by a range of local actors, are likely to be a vital aspect of localisation and adaptation of standardised outbreak response guidelines. The 2013–2016 West Africa Ebola outbreak provided clear evidence that local-level action can be significant in turning epidemics around. For example, citizens applied insights and past experience of disease control to protect themselves^15^ and to arrange safe burials and morally acceptable care of kin.^16^ Harnessing mobilisation, knowledge and inventiveness ‘on the ground’ and hearing from local people about their priorities, or concerns about other prevalent health and livelihood issues, is key to a proportionate and effective response that people understand and trust. Local organisations can document conditions, such as inequalities in availability of services, and provide access to a spectrum of information that can be vital for moving forward effectively. This can include epidemiologically relevant reports, given the lack of testing or recording of deaths in some settings. Localisation can also enable a response that is attentive to social differences and vulnerabilities.

### A typology of emerging locally adaptive responses

At this juncture, it is critical to share learning widely. To this end, we have distilled emerging domains for interventions, drawn from our knowledge and presented in a preliminary typology of adapted responses with localised examples (table 1). This table is not exhaustive but envisaged as an illustrative starter on a range of adapted alternatives, so that these can be documented and disseminated.

**Table 1:** Typology of Adaptive Responses with selected examples

Domain	Example of intervention
**Mitigating hardship**	
Livelihoods in the informal economy	Burkina Faso announced support for informal sector workers, as did Egypt and Tunisia. In Namibia, South Africa and Zimbabwe one-off or temporary cash grants have been provided to vulnerable people. Coverage depends on the strength of social protection systems and on access to mobile phones (for cash transfers), ID cards or inclusion in state databases. https://www.wiego.org/government-responses-covid-19-crisis
Food insecurity	Community Action Networks investigate the most appropriate ways to provide food relief for the hungry in Cape Town, South Africa. https://theconversation.com/local-networks-can-help-people-in-distress-south-africas-covid-19-response-needs-them-138219
Food markets	Urban municipal government in Bo, Sierra Leone, works with local researchers to identify hygiene and physical distancing measures in a busy open market to enable it to stay open safely. https://africanarguments.org/2020/03/30/preparing-for-covid-19-in-africa/.
**Care of the ill**	
Home care for COVID-19	Amref has partnered with governments to provide training on the safe provision and support of homecare for COVID-19 to community health workers via the Leap app, which works on simple mobile phones. https://newsroom.amref.org/blog/2020/08/building-health-worker-capacity-on-home-based-care-and-isolation-using-digital-learning-in-kenya/
**Protecting the medically vulnerable**	
Shielding the clinically vulnerable	Shielding is informally underway in Ethiopia, where community society groups and non-governmental organisations provide food, infection prevention and basic economic support to the shielded. https://gh.bmj.com/content/5/7/e003204.info
Maintaining essential healthcare	The International Community of Women Living with HIV West Africa has partnered with healthcare facilities to recruit a cadre of ‘community pharmacists’ to home deliver treatments for HIV and other conditions in Nigeria and Côte d’Ivoire. https://www.unaids.org/en/resources/presscentre/featurestories/2020/july/20200714_arv-cotedivoire-nigeria
**Preventing transmission**	
Local information	An existing network of residents has organised monitoring and ‘situation tracking’ of COVID-19 in informal settlements in Nairobi in order to liaise with the city government about service provision and the local situation, with assistance of community health volunteers. https://www.muungano.net/muunganos-covid-19-response
Dialogue and trust	In Kivu, DRC, members of the Amani Institute, a sociocultural movement, have worked with youth volunteers who visit public spaces and homes of internally displaced persons to share information about COVID-19 prevention. https://amani-institute.org/2020/05/04/coronavirus-in-a-context-of-conflicts-and-humanitarian-crisis-in-eastern-drcongo/
Burials	Traditional leaders in South Africa’s Eastern Cape have reintroduced *ukuqhusheka* or ‘secret burials’, a historic funerary practice with cultural significance that limits the number of people who attend bodies and burials. https://www.bbc.co.uk/news/world-africa-52571862
Isolation of those who are positive	Slum and Shack Dwellers International Kenya has provided input to the Department of Health’s guidelines for Isolation Centres, taking account of conditions in informal settlements. https://www.muungano.net/browseblogs/2020/6/11/covid-19-muungano-alliance-contributes-to-government-guidelines-on-isolation-centers
Screening, testing and contact tracing	In South Africa, cadres of community healthcare workers were deployed early for community-level screening and referral for testing, using skills gained in combating HIV and tuberculosis. https://www.ft.com/content/98d0d7c6-9bfb-4a64-bcab-19e0854a3b4d
Hygiene provision	Handwashing stations have been installed in places with limited water infrastructure, for example, informal settlements in Kenya and Rwanda. In Sudan, community committees made and distributed their own sanitisers. https://blogs.worldbank.org/nasikiliza/innovations-time-covid-19-rising-pandemics-challenges-africas-informal-settlements

### Supporting interconnections between state-led responses and mobilisation ‘on the ground’

A range of actors have been involved in localised interventions, including community-based and faith-based and other non-governmental organisations and the private sector, sometimes in partnership with central government, local authorities or international agencies. Other initiatives involve networks and federations, such as of informal settlement residents, or civil society groups. Furthermore, local organisation by those with informal authority can help in a crisis like COVID-19, as indeed occurred with Ebola in West Africa.^16^ Limitations in government capacities highlight the importance of coordinated activity between state responses and those ‘from below’, and interconnections and synergies between local experience and action, and government-led public health responses. The nature of existing governance structures can help determine which actors are best placed to coordinate efforts. Community-based actors with recognised local authority can act as key interlocutors and improve dialogue and trust.^12^

In arguing for adaptation and localisation, we are not proposing that the lion’s share of responsibility for preventing community transmission, mitigating socioeconomic impacts or protecting the vulnerable should fall on ‘communities’ or that responsibility to reduce infection risk should be construed as located with individuals alone. When extensive state-enforced restrictions are eased, it is easy for governments to resort to transferring responsibility for preventing infections to individuals or local leaders. This is especially problematic in contexts characterised by structural violence and where people’s living conditions make public health measures challenging to meet. It can lead to the blaming of groups and individuals who are marginalised, further intensifying inequalities that COVID-19 has laid bare. National, municipal and local authorities have important roles to play in partnering with community and grassroots groups to support and enable their efforts. Local-level action can play a further role in holding the state to account, if the political environment is favourable.

## Conclusion

Response plans for epidemics that privilege top-down action have been the norm, and governments have frequently echoed restrictive measures for COVID-19 as implemented in other global regions. For African countries, mounting evidence of deleterious effects of stringent approaches has led to growing acknowledgement of their limitations, and of the necessity for a diversity of responses, given also epidemiological heterogeneity. Various responses are emerging as decision makers in different countries adapt guidelines and pursue particular strategies, according to their social, economic and political contexts, as well as their histories and past disease experiences. Greater appreciation by scientists and policymakers of this diversity and its contextual shaping by factors that extend beyond epidemiology and public health need alone, is badly needed. We argue that it is also necessary to pay serious attention to initiatives that adapt measures at the local level, led by a range of actors and even emerging ‘from below’. This can assist in attuning interventions to different contextual realities and in ensuring that they are proportionate, attentive to vulnerabilities and social inequalities and socially just. We have shared some emergent positive examples that could be built on but this requires political will. These initiatives have been at the margins and urgently need to be brought in more centrally and given greater recognition and resourcing, both from national and international sources. Improving interconnections between responses ‘from above’ and ‘from below’ will be critical in moving forward.
